# Study on the shelf life and quality characteristics of highland barley fresh noodles as affected by microwave treatment and food preservatives

**DOI:** 10.1002/fsn3.1151

**Published:** 2019-07-30

**Authors:** Tuohetisayipu Tuersuntuoheti, Zhenhua Wang, Yanyan Zheng, Shuai Wang, Ziyuan Wang, Yan Wu, Shan Liang, Xinping Li, Min Zhang

**Affiliations:** ^1^ Beijing Advanced Innovation Center for Food Nutrition and Human Health Beijing Technology and Business University Beijing China; ^2^ Beijing Engineering and Technology Research Center of Food Additives Beijing Technology and Business University Beijing China

**Keywords:** highland barley fresh noodles, microwave, noodle quality, shelf life, ε‐poly‐L‐lysine

## Abstract

In this work, the effect of microwave (MW), ε‐poly‐L‐lysine (ε‐PL), calcium propionate (CP), and their combinations on the shelf life and quality characteristics of highland barley fresh noodle (HBFN) was studied. Firstly, the mixed flour was treated by MW (800 W) for different time. Subsequently, HBFN was prepared by mixing flour and sterilized water with addition of 0.04% ε‐PL combined with different concentrations of CP (0.025%, 0.020%, 0.015%) and stored at 25 ± 1°C. Changes of total plate count (TPC), moisture content, water state, textural properties, color, and pH in HBFN were monitored during storage. The results indicated that microwave treatment (40 s) provided 30% reduction of initial TPC of the flour without decline of noodle quality. Shelf life of HBFN treated with MW + ε‐PL (0.04%) + CP (0.02%/0.025%) reached 80 and 88 hr, respectively, and had good edible qualities during storage.

## 1 INTRODUCTION

Highland barley wet fresh noodle was obtained by mixing highland barley flour and wheat flour. It has a good freshness, unique flavor, and taste. Nowadays, with changing in dietary habits and the development of “functional” foods, there are increasing interests in searching for new foods, which contain functional ingredients (Narwal, Kumar, Sheoran, Verma, & Gupta, 2017). Highland barley is considered as the nutrition‐rich grain due to its high content of soluble fiber, β‐glucan, and antioxidants. Hence, it shows strong antioxidant activity and the ability to control cholesterol and blood glucose levels (Kim et al., 2007; Sullivan, Arendt, & Gallagher, 2013; Zhao et al., 2008). Therefore, highland barley products, as one of the functional foods, are attracting more and more researchers and consumers (Hatcher, Lagasse, Dexter, Rossnagel, & Izydorczyk, 2005; Holtekjølen, Bævre, Rødbotten, Berg, & Knutsen, 2008; Sharma & Gujral, 2014).

Due to the high initial microbe quantity, high moisture content, and abundant nutrition, highland barley fresh noodle is easy to rot. Hence, extending the shelf life of HBFN is one the crucial problems to be solved. So far, some methods such as irradiation, microwave, modified atmosphere packaging, and ozone treatment have been employed to extend the shelf life of fresh noodle products (Bai, Guo, Zhu, & Zhou, 2017; Klinmalai, Hagiwara, Sakiyama, & Ratanasumawong, 2017; Li, Zhu, Guo, Peng, & Zhou, 2011; Del Nobile et al., 2009; Zhang, Liu, Karim, & Ismail, 2018). Nevertheless, the initial microbe quantity in highland barley flour is very high at 5.4 lg CFU/g, while it is only 2 lg CFU/g in commercial wheat flour. Therefore, using only one physical preservation method (microwave) does not have an ideal effect, and for chemical preservative measures, a high‐level of addition is required in order to achieve an ideal fresh‐keeping effect, which is obviously not a green consumption. Therefore, finding an industrial used fence method that can inhibit and reduce microbial growth during storage is necessary.

Microwave treatment of food was used from 20th century (Fleming, 1944). But the argument on mechanism of microwave sterilization (whether it has nonthermal sterilizing effect or not) never ends; hence, theories about the nonthermal and thermal sterilizing effect have been established (Fung & Cunningham, 1980; Khalil & Villota, 1988; Sastry & Palaniappan, 2010). Microwave pasteurization and sterilization not only save the time, but also save the cots, as well as improve the quality of food (Maktabi, Watson, & Parton, 2011). Hence, it was used to different solid foods, such as egg yolk, pickled asparagus, and instant fresh noodles (Lau & Tang, 2002; Shenga, Singh, & Yadav, 2010). Considering the high initial microbe quantity in highland barley flour, microwave treatment was also used to reduce the microbes in the flour.

On the other hand, combination effect of ε‐poly‐L‐lysine (ε‐PL) and calcium propionate (CP) with different concentrations on shelf life extension and quality improvement of HBFN was investigated.

ε‐poly‐L‐lysine is a natural antibacterial peptide and decomposes into lysine in the human body, which can be used as a source of lysine (Chang, Lu, Park, & Kang, 2010). It was reported that ε‐PL could inhibit gram‐negative, gram‐positive bacteria, yeast, and molds (Badaoui, Kashtanov, & Chikindas, 2007; Geornaras & Sofos, 2005; Geornaras, Yoon, Belk, Smith, & Sofos, 2007; Hiraki, 2000). Due to its safety and strong antimicrobial activity against a wide spectrum of microorganisms, it is widely used in preservation of meat, fruits, vegetables, drinks, and milk products (Cheng, Wu, Yue, Wu, & Guo, 2008; Li et al., 2014; Min, Kwon, & Yoon, 2010; Qin & Zhou, 2006).

Calcium propionate is a new type food additive, and it can be absorbed by human body. It is a safe and reliable antimildew agent for food and feed approved by WHO and FAO (Cheng, 2009; Wei, 2002). Due to the wide range of antimicrobial effects against mold, yeast, and bacteria, CP is also used in preservation of meat, bread, hamburg, and fruits (Kwak, Kim, & Rhee, 2011; Ryan, Bello, & Arendt, 2008).

The concept of combining several preservation technologies, called “hurdle effect,” was recognized by Leistner and Gorris (1995). Accordingly, the purpose of this study was to reduce the initial microbe quantity in the mixed flour (highland barley flour and wheat flour) and inhibit the growth of microorganisms in HBFN by adding ε‐PL (0.04%) combined with different concentrations of CP, and at the same time, the shelf life and quality changes in HBFN were determined during storage at 25°C.

## 2 MATERIALS AND METHODS

### 2.1 Raw materials

High‐protein wheat flour (protein content 12.87% and moisture content 13.11%, respectively) was purchased from Yonghui supermarket, Beijing, China. There were two kinds of highland barley flour, which were extrusion flour and unextrusion flour. Unextrusion highland barley flour was obtained by grinding the highland barley grains using high efficiency pulverizer (Model WF‐20B, Keyi), and the extrusion highland barley flour was obtained by extruding unextrusion flour through twin screw extruder (Model SLG30‐6, Sabano) at the moisture content of 17% and grinding using WF‐20B high efficiency pulverizer. ε‐PL was purchased from Silive Biological Co., Ltd., Zhejiang, China. CP was purchased from Hengde Biotechnology Co., Ltd., Beijing, China. Other chemicals used in the experiment were of analytical grade. Highland barley flour was prepared according to highland barley extrusion modification method (Zhang, Liu, Tan, & Sun, 2016). The mixed flour was prepared at the ratio of 1:1:3 (w/w, highland barley extrusion flour, unextrusion flour, and wheat flour, respectively) based on the dry basis and then mixed thoroughly.

### 2.2 Microwave treatments

Of 100 g of the mixed flour was spread uniformly into the sterilization bag and then treated in a microwave oven (Model G80F20CN2L‐B8, Guangdong, China) at 800 W for 0, 10, 20, 30, 40, 50, and 60 s, respectively. Then, the reduction effect of microwave treatment on total plate count (TPC) and moisture content of mixture flour were investigated.

### 2.3 Determination of moisture content and TPC

The moisture content was determined following AACC method (Chemists, 2000). TPC was determined according to the food microbiological examination standard of China (GB4789.2—2016). Twenty‐five gram of the mixed flour or noodle samples were put into 225 ml 0.85% of aseptic physiological saline and then homogenized using a stomacher machine (Lab‐blender 400, Seward Laboratory) for 1 to 2 min. A series of tenfold dilutions were prepared, and 0.1 ml of the appropriate dilution was added into the total plate count agar plate to determine total microbe quantity. The plates were incubated at 37°C for 48 hr. All the experiments were run in triplicate.

### 2.4 Preparation of HBFN

Simulating industrial noodle making process, all the materials were wiped using 75% alcohol in advance. The HBFN was made of 200 g mixed flour, 81 ml distilled water with different concentrations of ε‐PL and CP, and 2 g salt (NaCl). A dough maker was used to prepare noodle crumbles (Model JHMZ‐200, Dongfujiuheng, China). The speed of dough maker was 90 rpm, and mixing time was 8 min. The obtained dough crumbles were passed through the roller unit of experimental pasta machine (Model JMTD‐168/140, Dongfujiuheng, China) with the roller gap of 1.5 mm for three times, then placed into a sterilized plastic bag, and rested for 30 min at 30°C. Then, the dough sheet was pressed through the pasta machine with the roller gap gradually reduced from 2.5 to 1.0 mm to get the dough sheet with 1.0 mm in thickness. Subsequently, the dough sheets were cut into strands with 20 cm in length and 1.5 mm in width and put into the plastic bags. For the noodles, the addition ratio of ε‐PL and CP includes ε‐PL (0.04%) + CP (0.015%), ε‐PL (0.04%) + CP (0.020%), and ε‐PL (0.04%) + CP (0.025%), respectively.

### 2.5 Textural analysis

Before testing textural properties, the optimal cooking time (OCT) of HBFN was measured according to the method described by Bai et al. (2017). Briefly, 25 g of HBFN was cooked in 500 ml of boiling distilled water. The cooked noodles were squeezed by two transparent glass plates until the central white core disappeared. The time it takes to disappear the white core was termed as OCT. The textural properties of HBFN were measured by texture analyzer (Model TMS‐Pilot, FTC) according to the OCT under the parameters as follows: compress was 70%; pretest, test, and post‐test speed was 0.8 mm/s; interval time was 1 s; and the load was 0.05 N.

### 2.6 Determination of water state

The water state of fresh noodle sheets was analyzed with a LF‐NMR system (Model NMI‐20, Niumag). The transverse relaxation time *T*_2_ was determined using a CPMG pulse sequence. Uncooked HBFN was scanned at 32 ± 0.01°C. The number of sampling points (TD) was 10,106, number of echo (NECH) was 500, TW was 1,000, and repeated time was 32. Each sample was scanned in triplicate.

### 2.7 Determination of pH

Uncooked HBFN sample (10 g) was put into 90 ml of distilled water and homogenized by stomacher machine (Lab‐blender 400, Seward Laboratory) for 2 min and obtained the uniform mixture. A pH meter (Model PHSJ‐3F, Inesa Analytical Instrument) was used to measure the Ph of HBFN.

### 2.8 Determination of color

HBFN sheets were cut into pieces of approximately 5 cm in width and length, and placed in the plastic bags at 25°C. The color was measured every 12 hr used a chroma meter (Model CR‐400, KONICA MINOLTA) with the CIE 1976 L*, a*, and b* color scale.

### 2.9 Statistical analysis

Analysis of variance (ANOVA) was performed using Duncan's multiple range test by SPSS version 20.0, and significant differences were defined at *p* < .05. All tests were represented as the mean of three replicates and standard deviation (*SD*). TPC data in figures were transformed into logarithms of the number of colony forming units (CFU/g).

## 3 RESULTS AND DISCUSSION

### 3.1 Changes of initial TPC and moisture content in mixed flour treated by microwave

**Figure 1:**
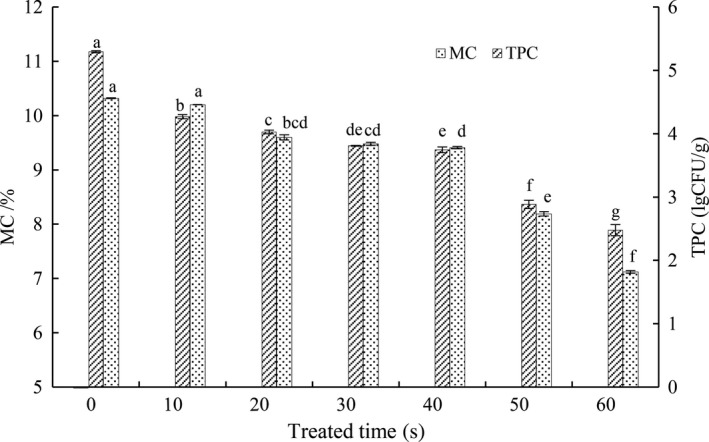
Changes of TPC and MC in flour after different treated time. Different characters (a–g) on the top of the same columns indicate significant difference at *p* < .05 level in moisture content and total palte count of mixed flour among different MW treatment

The high level of initial microbes in raw materials (flour and water) is considered as the most important factor leading to the perishability of fresh noodles (Li, Luo, et al., 2012; Li, Zhu, et al., 2012). Microwave treatment of flour has been shown to decrease the number of microorganisms (Zhou, Liu, Wu, & Kang, 2016). Changes in initial TPC and moisture content in mixed flour after microwave treatment are shown in Figure 1. With the increase in treated time, there was a significant (*p* < .05) reduction in initial TPC of the mixed flour, especially from 30 to 60 s. Considering from the point of TPC reduction, 60 s was chosen for microwave treatment sterilization. However, it should be noted that the temperature of mixed flour was increased rapidly with the microwave‐treated time and then appeared serious caking phenomenon in 60 s. It had been investigated that there was a strong correlation between the caking phenomenon and temperature (Aguilera, Valle, & Karel, 1995). High‐power or long‐time microwave treatment also destroyed the intramolecular and intermolecular hydrogen bonds of amylose and degraded amylopectin molecules. Thus, it would reduce the gelatinization temperature, accelerate water absorbed gelatinization of wheat flour, and increase viscosity (Mei, Xiong, & Qingjie, 2013). Besides, moisture content decreased with the treated time, especially in 50 and 60 s. Therefore, the time of 60 s was rejected during the microwave treatment.

### 3.2 Changes of textural quality and color in HBFN with microwave treatment

**Figure 2:**
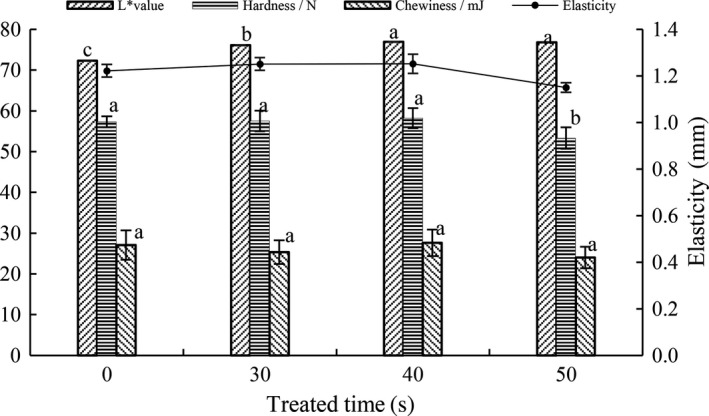
Changes of hardness, elasticity, chewiness, and L* value in HBFN. Different characters (a–c) on the top of the same columns indicate significant difference at *p* < .05 level in textural properties and L* value of HBFN among different MW treatment

To determine the optimum treated time, the textural properties and color changes in HBFN by different treated times (30, 40, 50 s) were investigated Figure 2. Treated time of 30 and 40 s could improve the elasticity and color of noodles, especially for 40 s, and the other properties were not significantly affected compared with the control. Fifty seconds could only improve the color of noodles, but had negative effect on hardness, elasticity, and chewiness. Hence, microwave treatment could improve the noodle quality and color, but long‐time and high‐power microwave processing would decline the texture profile, which was accordance with other researches (Yu, Chen, & Zhou, 2017; Zhang et al., 2018; Zhou et al., 2016).

Relatively speaking, 10 and 20 s were not very effective on reducing initial TPC. Fifty seconds and sixty seconds had a good effect on reducing initial TPC, but both flour quality and noodle quality were also declined. Therefore, in terms of bactericidal efficiency and noodle quality, the optimum microwave treatment time of flour was 40 s.

### 3.3 Changes of TPC in HBFN with different treatment during storage at 25°C

**Figure 3:**
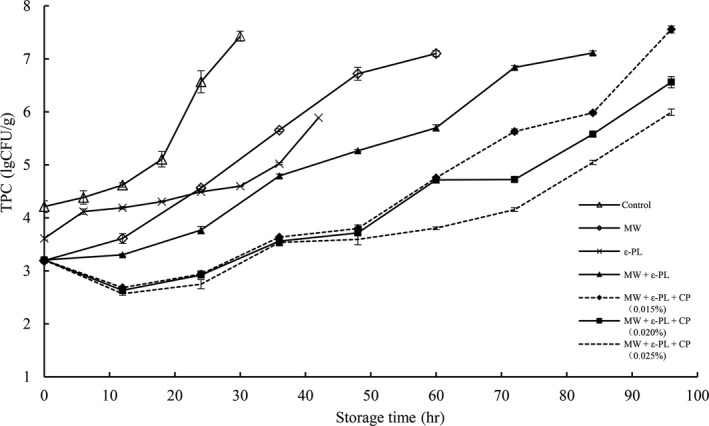
Total plate count changes in HBFN with different treatment during storage at 25°C

According to the Chinese standard of NY/T1512‐2014 “green food—uncooked processed food made of cereals,” the TPC value of 3 × 10^5^ CFU/g was defined as the upper limit of microbe in fresh noodles. Hence, it is terminated to test the microbe quantity when TPC overtake 3 × 10^5^ CFU/g, and changes of TPC in HBFN with different treatment during storage at 25°C are showed in Figure 3. It can be seen that the TPC in newly produced HBFN samples and microwave‐treated samples (40 s) were 4.2 and 3.2 lg CFU/g, respectively. However, microwave treatment could not completely kill the microorganism in mixed flour, and with the extension of storage time, the viable microorganism would multiply. Therefore, compared with the shelf life of control of 19 hr, ε‐PL (0.04%) and microwave treatment only extended the shelf life of HBFN to 38 and 32 hr, while MW + ε‐PL (0.04%) + CP (0.015%), MW + ε‐PL (0.04%) + CP (0.020%), and MW + ε‐PL (0.04%) + CP (0.025%) extend the shelf life of HBFN to 68, 80, and 88 hr, respectively. The results indicated that combination of MW, ε‐PL, and CP had a good “hurdle effect” on both reducing and inhibiting microorganisms, which were in agreement with the results Li et al. (2014), Ma, Cheng, Li, and Yang (2014), Wu (2015), Zhang et al. (2018) and Zhou et al. (2016). The sterilization effect of MW could kill all kinds of microorganisms in a short time, and ε‐PL and CP had strong antimicrobial activity against a wide spectrum of microorganisms in food. Therefore, synergetic effect would be observed if they were combined. Considering from the trend of “green” consumerism, the effect on shelf life and quality improvement of HBFN, subsequent investigations were carried out on the fresh noodles with MW + ε‐PL (0.04%) + CP (0.020%) and MW + ε‐PL (0.04%) + CP (0.025%).

### 3.4 Changes of textural properties in cooked HBFN with different treatment during storage at 25°C

The texture of noodles is the most important issue for consumers (Ajila, Aalami, Leelavathi, & Rao, 2010). Changes of textural parameters in HBFN during storage are showed in Table 1. All samples generally had a downward trend in hardness, elasticity, and chewiness, but the treated groups appeared to be higher in all these parameters. Compared with the control, the decrease in hardness and elasticity was not significant within the shelf life. It was reported that protein content (glutenin content and gliadin content) affected the hardness, elasticity, and chewiness of noodles (Lu, 2010). It formed a rigid and elastic network structure by intermolecular disulfide bonds and secondary bonds (such as hydrogen bonds and hydrophobic interactions) between subunits of glutenin. Hence, the higher the glutenin content, the better the hardness and elasticity of the noodle. Gliadin changes the rheological properties and processing properties of dough by interfering with or changing the relative content of different types of glutenin (Feillet, Ait‐Mouh, Kobrehel, & Autran, 1989). In control group, because of the quick microbial proliferation, the destruction of gluten network of HBFN is serious, which would lead to the reduction of hardness, elasticity, and chewiness. In treated groups, the antimicrobial agents delayed the destruction of gluten protein by inhibiting the growth of microbes. Besides, there was a slight increase in hardness during storage (eg: 36 hr, in control), which might be related to the water loss (Hu, Wei, & Chen, 2017). Additionally, due to the water permeability of plastic bags, the water may diffuse to the surrounding atmosphere and led to the increase in hardness (Bai et al., 2017).

**Table 1:** Changes in textural properties of HBFN during storage

Parameters	Storage time/h	Hardness/*N*	Elasticity/mm	Chewiness/mJ
Control	0	59.72 ± 1.04^ab^	1.14 ± 0.03^a^	35.60 ± 0.51^a^
	12	58.64 ± 0.14^a^	1.02 ± 0.02^bc^	30.35 ± 1.42^cd^
	24	57.61 ± 0.65^ab^	0.92 ± 0.15^abc^	29.90 ± 1.46^d^
	36	59.85 ± 0.34^a^	1.03 ± 0.01^abc^	31.36 ± 1.01^bcd^
	48	55.23 ± 0.45^c^	0.89 ± 0.03^c^	22.00 ± 0.58^e^
Microwave + ε‐PL (0.04%) + CP（0.020%)	0	63.56 ± 0.55^a^	1.36 ± 0.01^a^	41.94 ± 0.83^ae^
	12	60.16 ± 0.75^cde^	1.10 ± 0.04^bcdef^	30.84 ± 0.91^bcdef^
	24	59.91 ± 1.00^de^	1.04 ± 0.03^ghi^	28.34 ± 2.37^ef^
	36	61.89 ± 1.55^abcd^	1.09 ± 0.05^cdefg^	30.41 ± 0.86^cdef^
	48	60.92 ± 1.60^abcde^	1.05 ± 0.05^fghi^	28.90 ± 3.75^abcdef^
	60	60.38 ± 2.34^bcdef^	1.06 ± 0.05^efghi^	28.50 ± 2.92^abcdef^
	72	56.32 ± 1.28^g^	1.01 ± 0.03^hi^	26.17 ± 0.57^f^
	84	58.90 ± 2.66^efg^	1.07 ± 0.01^defgh^	29.02 ± 0.87^def^
	96	56.90 ± 1.35^fg^	1.00 ± 0.04^i^	23.15 ± 2.27^abcdef^
Microwave + ε‐PL (0.04%) + CP（0.025%)	0	59.86 ± 0.45^a^	1.17 ± 0.05^a^	35.02 ± 1.913^a^
	12	59.56 ± 0.79^a^	1.15 ± 0.02^a^	30.72 ± 0.85^bcdef^
	24	59.55 ± 0.61^a^	1.04 ± 0.02^e^	29.00 ± 0.57^cdefghi^
	36	58.86 ± 1.27^a^	1.01 ± 0.04^f^	28.18 ± 1.61^efghi^
	48	58.11 ± 1.05^a^	1.05 ± 0.01^def^	27.09 ± 1.57^ghi^
	60	59.07 ± 1.57^a^	1.05 ± 0.07^cdef^	28.64 ± 3.84^defghi^
	72	59.24 ± 1.33^a^	1.01 ± 0.03^f^	26.65 ± 1.16^i^
	84	58.27 ± 2.99^a^	1.06 ± 0.03^bcdef^	27.88 ± 2.36^fhig^
	96	57.84 ± 0.95^a^	1.05 ± 0.02^def^	26.85 ± 2.70^hi^

Note

Means in the column with different small superscript letters indicate significant difference at *p* < .05

### 3.5 Changes of water state in HBFN with different treatment during storage at 25°C

**Figure 4:**
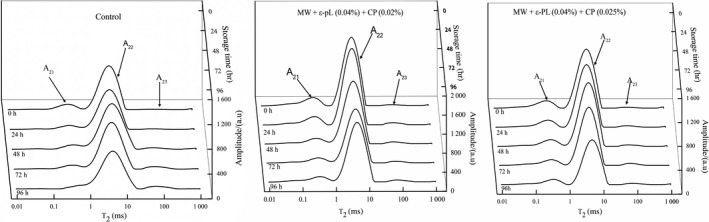
*T*_2_ value in HBFN with different treatment during storage at 25°C

Water can interact with other components of food, which resulted in affecting the quality and stability of food (Carini, Vittadini, Curti, Antoniazzi, & Viazzani, 2010). Water is considered a vital index to measure the quality of foods (Li, Luo, et al., 2012; Li, Zhu, et al., 2012). Hence, according to the state (bound or free) of water molecules in fresh noodles, we can measure the quality changes of HBFN during storage. Water inversion results of transverse relation times *T*_2_ and the related peak area in HBFN with different treatment during storage are shown in Figure 4 and Table 2.

**Table 2:** Changes of transverse relaxation time *T*_2_ in HBFN with different treatment during storage at 25°C

Parameters	Storage time/h	*T*_21_ (ms)	A_21_ (%)	*T*_22_ (ms)	A_22_ (%)	*T*_23_ (ms)	A_23_ (%)
control	0	0.43 ± 0.03^ab^	10.21 ± 0.19^a^	5.34 ± 0.00^a^	88.14 ± 0.28^e^	77.43 ± 0.00^a^	0.58 ± 0.03^d^
	24	0.41 ± 0.00^ab^	7.99 ± 0.46^b^	5.34 ± 0.00^a^	89.16 ± 0.67^cd^	68.93 ± 0.00^b^	1.18 ± 0.13^cd^
	48	0.47 ± 0.05^a^	6.46 ± 0.07^cd^	4.75 ± 0.00^b^	89.76 ± 0.44^bc^	68.93 ± 0.00^b^	2.35 ± 0.32^bcd^
	72	0.37 ± 0.08^b^	6.40 ± 0.28^d^	4.23 ± 0.00^c^	88.59 ± 0.27^de^	68.93 ± 0.00^b^	4.02 ± 0.17^ad^
	96	0.04 ± 0.02^c^	0.25 ± 0.22^e^	4.23 ± 0.00^c^	94.66 ± 0.22^a^	61.36 ± 0.00^c^	4.95 ± 0.17^a^
MW + ε‐PL (0.04%) + CP（0.020%)	0	0.37 ± 0.00^a^	12.58 ± 0.23^a^	4.95 ± 0.33^a^	86.09 ± 0.17^d^	77.43 ± 0.00^a^	1.11 ± 0.10^e^
	24	0.34 ± 0.02^a^	10.60 ± 0.26^b^	4.75 ± 0.00^b^	87.32 ± 0.31^c^	77.43 ± 0.00^a^	1.65 ± 0.14^d^
	48	0.35 ± 0.02^a^	9.48 ± 0.79^cd^	4.75 ± 0.00^b^	88.45 ± 0.56^ab^	68.93 ± 0.00^b^	1.97 ± 0.07^c^
	72	0.35 ± 0.02^a^	8.42 ± 0.46^e^	4.23 ± 0.00^c^	88.91 ± 0.23^a^	68.93 ± 0.00^b^	2.00 ± 0.44^b^
	96	0.33 ± 0.00^b^	9.08 ± 0.69^de^	4.23 ± 0.00^c^	88.06 ± 0.42^b^	68.93 ± 0.00^b^	2.30 ± 0.29^a^
MW + ε‐PL (0.04%) + CP（0.025%)	0	0.37 ± 0.00^a^	12.17 ± 0.00^a^	5.34 ± 0.00^a^	86.61 ± 0.00^b^	77.43 ± 0.00^a^	0.87 ± 0.00^c^
	24	0.33 ± 0.00^b^	10.21 ± 0.24^ce^	4.75 ± 0.00^b^	87.76 ± 0.05^a^	68.93 ± 0.00^b^	1.72 ± 0.13^bc^
	48	0.38 ± 0.00^a^	9.50 ± 0.36^de^	4.75 ± 0.00^b^	88.57 ± 0.19^a^	68.93 ± 0.02^b^	1.56 ± 0.28^abc^
	72	0.33 ± 0.00^b^	10.29 ± 0.00^bc^	4.23 ± 0.00^c^	87.36 ± 0.00^a^	68.93 ± 0.00^b^	2.35 ± 0.00^ab^
	96	0.33 ± 0.00^b^	9.36 ± 0.27^e^	4.23 ± 0.00^c^	88.26 ± 0.35^ab^	68.93 ± 0.00^b^	2.37 ± 0.10^a^

Note

Means in the column with different small superscript letters indicate significant difference at *p* < .05

Three populations of *T*_2_ were measured in HBFN samples. It indicated the presence of multiple water domains in the noodle samples. The results were consistent with the previous studies (Hu et al., 2017; Kontogiorgos, Goff, & Kasapis, 2008; Li, Luo, et al., 2012; Li, Ma, Zhu, Guo, & Zhou, 2017; Li, Zhu, et al., 2012). In all samples, an increased peak area percentage was observed between 10 and 100 ms in the end of storage, especially in the control group, which indicated that more flowing water is released due to the broken structure and the weakened water–solid interaction (Li et al., 2017).

*T*_2_ value reflects the mobility of water in HBFN. The strongly bound water *T*_21_, weakly bound water *T*_22_, and free water *T*_23_ in the HBFN showed a decreasing trend with the storage time. Considering the forms of water proportion, the weakly bound water peak area percentage A_22_ and free water peak area percentage A_23_ had tendency to increase, while the strongly bound water peak area percentage A_21_ decreased, especially in control group that the strongly bound water peak area percentage declined from 10.21% at the beginning to 0.25% in the end of storage, while treated groups declined from 12.58%, 12.17% to 9.08% and 9.36%, respectively. The strongly bound water gradually shifted to the weakly bound water and free water, which is mainly caused by the deterioration of HBFN. In control groups, the proliferation of microbes accelerated the spoilage of noodles and weakened the combination of water and non‐aqueous components, while it inhibited the microbial growth and delayed the destruction of noodle structure, in treated groups. The results were consistent with the results reported Hu et al. (2017) and Li et al. (2017).

### 3.6 Changes of moisture content and pH in HBFN with different treatment during storage at 25°C

**Figure 5:**
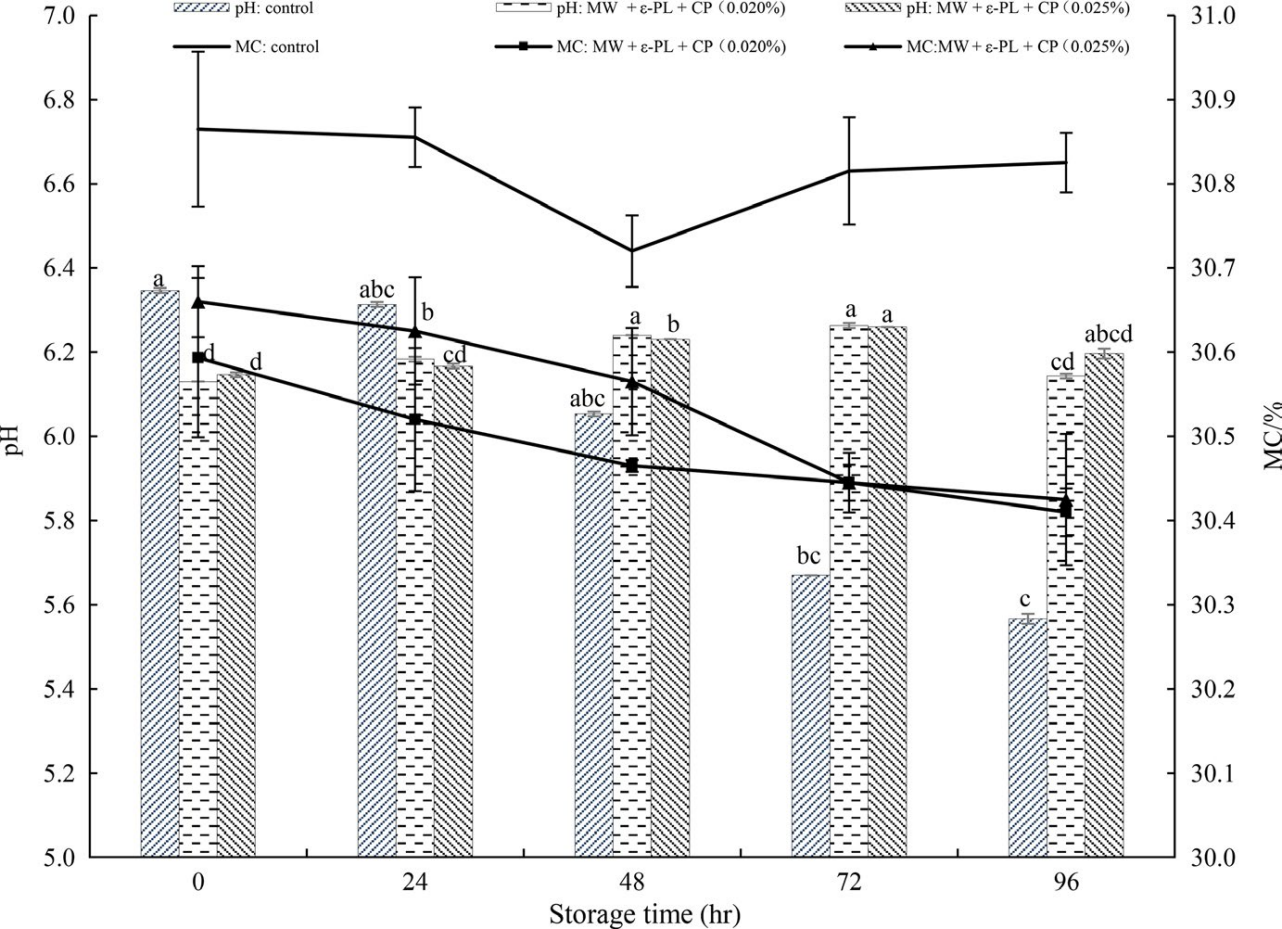
Changes of moisture content and pH in HBFN with different treatment during storage at 25°C. Different characters (a–d) on the top of the same columns indicate significant difference at *p* < .05 level in pH value of HBFN during different storage time

The pH and moisture content changes in HBFN during storage are presented in Figure 5. The initial pH value of HBFN was 6.35, 6.13, and 6.15 respectively, which was suitable for the growth of microorganism. For the control group, the pH values declined continuously, especially from 48 to 72 hr, and then, the decline was slight in the end of storage (the rate of decline slowly and stabilized). It may due to the fermentation, which is one of the main spoilages for fresh noodles. The noodle components (mainly carbohydrates) can be used by microorganisms and produce acids; hence, it is common to decrease the pH value during storage (Ghaffar, Abdulamir, Bakar, Karim, & Saari, 2009). Besides, with the spoilage of HBFN, proteins could be break down to some small molecular compounds, such as amino acids. These compounds interact with the acids produced by microorganisms and lead to the stabilization of pH value (Li & Han, 2010). For the treated groups, they were relatively low at the beginning of storage, increased from 24 hr, and then decreased in the last period of storage. That is because, the pH value of 5% ε‐PL solution and diluted solution is 4.13 and 5.98 at 25°C, while CP solution is weakly alkaline. The two solutions (ε‐PL and CP) might take an acid and base neutralization reaction, when they were mixed, which lead to the pH relatively low at the beginning of storage. The additives were consumed and pH increased during storage, but the pH value decreased again due to microbial fermentation in the end of storage. Hence, the pH changes during storage are remained to be further studied.

### 3.7 Changes of color in HBFN with different treatment during storage at 25°C

**Figure 6:**
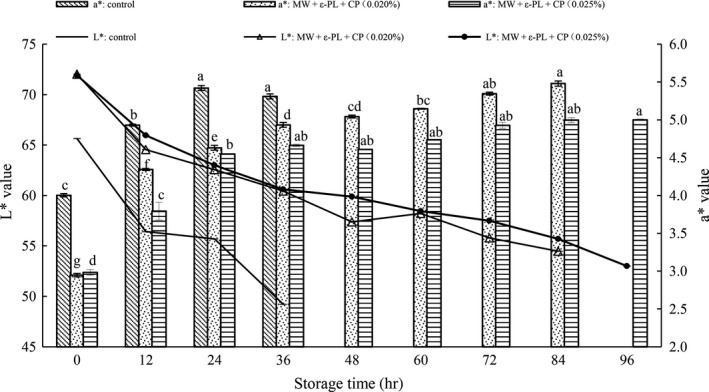
Color changes in HBFN with different treatment during storage at 25°C. Different characters (a–g) on the top of the same columns indicate significant difference at *p* < .05 level in a* value of HBFN during different storage time

Color is considered as one of the major factors of fresh noodle marketability, and one of the important factors affecting on the quality of HBFN during storage. As shown in Figure 6, with the extension of storage time, L* value decreased and a* value increased, especially in the first 12 hr. The decrease in L* value of fresh noodles was highly significant during the 24 hr, especially during the first 4 hr (Li et al., 2017). The initial L* value of control and treated groups were 65.64, 72.20, and 71.91 respectively. The decrease in L* value and the increase in a* value of HBFN were mainly due to the oxidant browning of polyphenol oxidase in the mixed flour (Asenstorfer, Appelbee, & Mares, 2009; Hu et al., 2017). Polyphenols were oxidized by polyphenol oxidase and formed color substances. The treated group flour was treated with microwave, which could reduce the activity of polyphenol oxidase (Yu et al., 2017; Zhou et al., 2016). In addition, with the prolongation of storage time, the loosening of network structure not only reduced the reflectivity, but also made the polyphenol oxidase reaction easier (Ong, Ross, & Engle, 2010), which decreased the L* value. Besides, it can be seen from the diagram that the L* value of the noodles in treated group also decreased continuously during the storage. Therefore, it should be considered not only the inhibition of microorganisms, but also the inhibition of the discoloration through applying ant browning agents, such as citric acid, lemon juice, and white wine (Brütsch et al., 2018), and it remains to be further study in subsequent study.

## 4 CONCLUSION

The present study indicated that microwave treatment (800 W) for 40 s provided 30% reduction of initial TPC of the mixed flour without decline of noodle quality. Shelf life of HBFN treated with MW + ε‐PL (0.04%) + CP (0.020%/0.025%) reached 80 and 88 hr, respectively, while HBFN had good edible and color qualities during storage at 25°C. The results point out that the “hurdle effect” is a practical and economical approach, promising technique to prolong the shelf life of highland barley fresh noodles.

## CONFLICT OF INTEREST

The authors have no conflict of interest to declare.

## ETHICAL APPROVAL

This article does not involve any human or animal testing.

## INFORMED CONSENT

None.
